# Exploiting the interplay between cross-sectional and longitudinal data in Class III malocclusion patients

**DOI:** 10.1038/s41598-019-42384-7

**Published:** 2019-04-17

**Authors:** Enrico Barelli, Ennio Ottaviani, Pietro Auconi, Guido Caldarelli, Veronica Giuntini, James A. McNamara, Lorenzo Franchi

**Affiliations:** 1 OnAIR Ltd, Genoa, Italy; 2 Department of Mathematics, Università degli Studi di Genova, Genoa, Italy; 3 Private Practice of Orthodontics, Roma, Italy; 4 IMT School for Advanced Studies, Piazza San Francesco 19, 55100 Lucca, Italy; 5 Istituto dei Sistemi Complessi CNR, Unità Sapienza, Dip. Fisica, P.le A. Moro 2, 00185 Rome, Italy; 6 ECLT, San Marco 2940, 30124 Venice, Italy; 7 Department of Experimental and Clinical Medicine, Orthodontics, Università degli Studi di Firenze, Florence, Italy; 8 Department of Orthodontics and Pediatric Dentistry School of Dentistry, University of Michigan, Ann Arbor, Michigan USA; 9 School of Medicine and Center for Human Growth and Development, The University of Michigan, Ann Arbor, Michigan USA

**Keywords:** Data processing, Complex networks

## Abstract

The aim of the study was to investigate how to improve the forecasting of craniofacial unbalance risk during growth among patients affected by Class III malocclusion. To this purpose we used computational methodologies such as Transductive Learning (TL), Boosting (B), and Feature Engineering (FE) instead of the traditional statistical analysis based on Classification trees and logistic models. Such techniques have been applied to cephalometric data from 728 cross-sectional untreated Class III subjects (6–14 years of age) and from 91 untreated Class III subjects followed longitudinally during the growth process. A cephalometric analysis comprising 11 variables has also been performed. The subjects followed longitudinally were divided into two subgroups: favourable and unfavourable growth, in comparison with normal craniofacial growth. With respect to traditional statistical predictive analytics, TL increased the accuracy in identifying subjects at risk of unfavourable growth. TL algorithm was useful in diffusion of information from longitudinal to cross-sectional subjects. The accuracy in identifying high-risk subjects to growth worsening increased from 63% to 78%. Finally, a further increase in identification accuracy, up to 83%, was produced by FE. A ranking of important variables in identifying subjects at risk of growth worsening, therefore, has been obtained.

## Introduction

Data Mining and Machine Learning are mathematical procedures focused on developing algorithms and logical statements that can learn from the data and make subsequent predictions^1–3^. These algorithms have been used successfully to extract meaningful information highlighting important, potentially unintuitive system behaviours in various domains such as econometrics, aerospace, robotics, finance, weather forecasting, and biomedicine^3,4^. In medicine, research on computer-aided diagnosis from artificial intelligence began to be applied with hopes that difficult clinical diagnostic and prognostic problems might yield to mathematical formalism, and that computers might have active role as consultants^5^. Over the years it became clear that whenever the process is not solely stochastic, machine-learning algorithms could provide some insight into the likelihood of which outcome will occur (the odds, not the absolute certainty) by identifying new, high-order concepts and relations within components of biomedical pathways and generating automated hypotheses within syndromes^6^. Important tasks in bioinformatics are the prediction of a response variable (i.e., disease status of a patient), based on a large number of predictors (i.e., signs and symptoms), and the reliable identification of the relevance of each candidate variable to patient outcome. Although Machine Learning tools have been developed to decipher the underlying patterns in thousands or millions of data, in the medical field these methods also have provided useful prognostic indications based on characteristics from a few dozen patients^5–9^.

Malocclusions are isoforms of disharmony: they incur costs in terms of weakness of mechanotransduction, cumulative occlusal trauma, and outcome uncertainty about the ultimate facial appearance^9^. Class III malocclusion is characterised by the protrusion of the lower dental arch and incorrect relation between the teeth of the two dental arches when they approach each other as the jaws close. The contemporary approach to treatment of Class III malocclusion is aimed to avoid orthognatic surgery and potential complications. The necessity at growth modification promoting mandibular restraining, maxillary growth, and dentoalveolar compensation has been particularly stressed^9^. Therefore it would be highly desirable at the early treatment planning to have knowledge of complex morphological, growth, and function characteristics rendering any individual patient as either favorable or unfavorable for successful orthodontic treatment.

The contemporary approach to treatment of Class III malocclusion is aimed to avoid orthognathic surgery and potential complications. The necessity at growth modification promoting mandibular restraining, maxillary protraction, and dentoalveolar compensation has been particularly stressed^9^. Therefore, it would be highly desirable at the early treatment planning to have knowledge of complex morphological, growth, and function characteristics rendering any individual patient as either favorable or unfavorable for successful orthodontic treatment.

Class III disharmony may show a significant tendency to worsen with growth if a patient is left untreated. Moreover a discrepancy between maxillary and mandibular growth can result in relapse of the malocclusion after the pubertal growth peak, and some patients may need corrective surgery at the end of craniofacial growth. Accordingly, the ability to predict the outcome of this disharmony would provide a significant advantage in orthodontic diagnosis and prognosis.

In clinical orthodontics traditional statistical models suffer from holding categorical data, correlate variables, missing values, and scarce amount of patient’s longitudinal data from which to derive the quality of future craniofacial growth and treatment outcomes. On the contrary, large numbers of subjects with unlabelled growth outcomes (i.e. cross-sectional subjects) are readily available, though there are few ways to use these subjects for growth prediction^9^. Only in longitudinal surveys are orthodontic researchers able to detect changes in the growth characteristics of the target population, at both group and individual levels. As a result, they can establish sequences of growth events. Moreover, cross-sectional studies typically do not provide definitive information about cause-and-effect relationships. Longitudinal data on untreated subjects with Class III malocclusion are rare, given the functional and aesthetic challenges. As the skeletal imbalance usually tends to get worse over time, individuals with this condition usually face treatment during childhood and adolescence. The intriguing characteristic of a Transductive Learning (TL) procedure (also known as semi-supervised learning) resides in its capability of generating additional information derived from labeled (longitudinal) data through the spread of information to unlabelled (cross-sectional) data, i.e. subjects whose outcome is unknown^10–15^. Therefore, TL algorithms take advantage of unlabelled data, when used in conjunction with a small amount of labeled data, to produce considerable improvement in prediction accuracy. In each step of this methodology, a subset of unlabelled cross-sectional subjects is labeled according to an iterative expectation-maximisation algorithm^15^. Recently, Auconi *et al*.^16^ applied two commonly used statistical methods, *Classification Trees* (CT) and *Discriminant Analysis* (DA), to forecast the individual risk of unfavourable dentoskeletal growth among a particular set of untreated subjects affected by Class III malocclusion. Among 91 subjects, Auconi and coworkers found 28 “good growers” and 63 “bad growers”^16^. Two cephalometric angles (SNA and PPMP see below) revealed to be strongly related to the risk of unfavorable growth. However, the ability of these methods to predict the risk among new, unseen Class III subjects was relatively modest (accuracy: 64%). The purpose of this study is to submit the cephalometric values of this same dataset to a sequence of procedures such as *transductive learning*, *boosting*, *and feature engineering*, with the aim to improve the interpretability of the growth model and the prediction of risk of unfavorable dentoskeletal prognosis in these subjects.

## Material and Methods

Two samples of longitudinal and cross-sectional untreated Class III subjects were analysed. The longitudinal sample consisted of 91 untreated Class III subjects (48 females and 43 males) who were enrolled in the Auconi’s study^16^. Two lateral cephalograms were available for each subject. The mean age at *T*_1_ was 10.4 ± 2.0 years while the mean age at *T*_2_ was 15.4 ± 1.9 years. The cross-sectional sample comprised 728 untreated Class III subjects (341 males and 387 females) with an age range from 7 to 13 years. This sample was enrolled previously in descriptive estimates of craniofacial growth in Class III malocclusion^9,16–19^. To be included in this study, the subjects of both samples had to satisfy the following criteria: Caucasian ancestry, no previous orthodontic treatment, no congenitally missing teeth, and no craniofacial syndromes. Diagnosis of Class III malocclusion was based on accentuated mesial step relationship of the primary second molars, anterior crossbite, permanent first molar relationship of at least one-half cusp Class III, Wits appraisal less than −2 mm, ANB angle less than 0 degree. This study was exempted from review by the Medical School Institutional Review Board of the University of Michigan, Ann Arbor (HUM00143467).

### Cephalometric analysis

**Figure 1:**
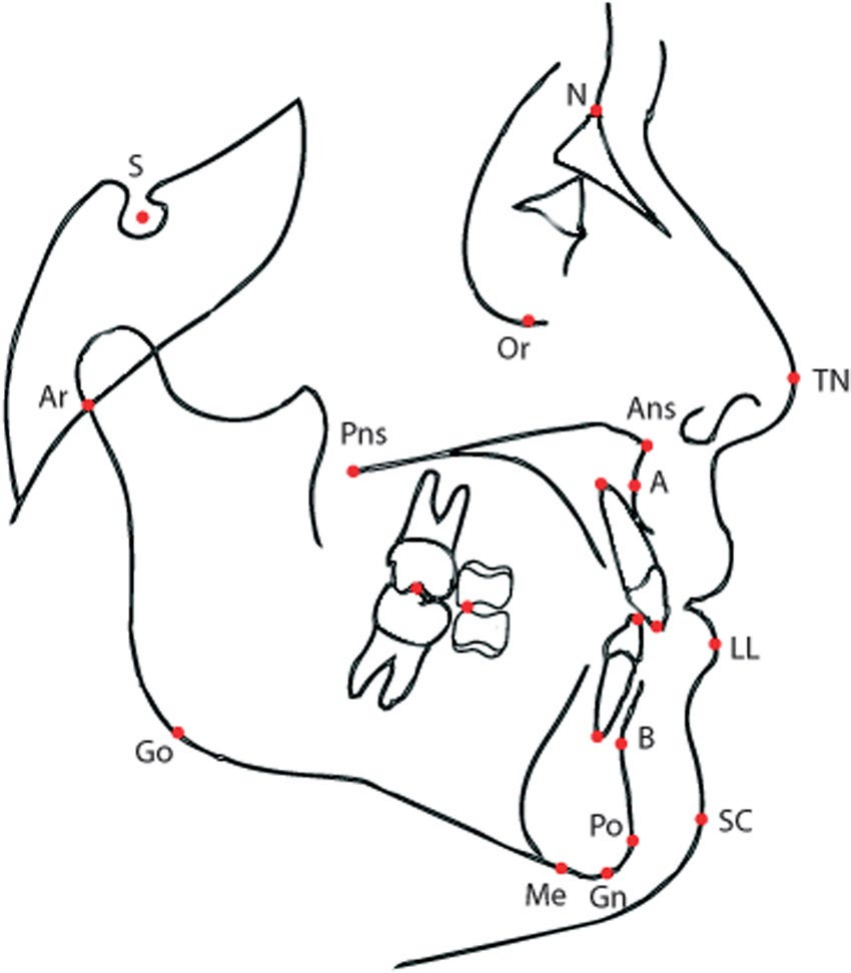
Cephalometric landmarks.

The quantities under consideration (as in similar analyses^16^) are derived from the anatomy of the patient as shown in Fig. 1. They are based on the following 11 variables: *S*–*N*, *S*–*Ar*, *NSAr*, *SNA*, *SNB*, *Co*–*Go*, *Go*–*Gn*, *N*–*Me*, *ArGoMe*, Palatal Plane to *S*–*N* (*PPSN*) and Palatal Plane to Mandibular Plane (*PPMP*) defined below. In particular we have the following quantities with two measurements at ages *T*_1_ and *T*_2_ (measured in years):

- *ID*: anonymised ID code unique to each patient.
- *Growth*: a binary variable with values “Good” or “Bad”, determined on the basis of CoGn-CoA.
- *S*-*N*: length of the anterior cranial base (Sella-Nasion) (mm).
- *S*-*Ar*: distance from point Sella to point Articulare (mm).
- *NSAr*: Saddle angle (Nasion-Sella Articulare) (degrees).
- *SNA*: antero-posterior position of the maxilla to the anterior cranial base (degrees).
- *SNB*: antero-posterior position of the mandible to the anterior cranial base (degrees).
- *ANB*: angle between Down’s points A and B (degrees).
- *PPSN*: palatal plane to anterior cranial base angle (degrees).
- *PPMP*: palatal plane to mandibular plane angle (degrees).
- *N*-*Me*: total anterior face height (mm).
- *Co*-*A*: maxillary length from Condylion to point A (mm).
- *Co*-*Gn*: total mandibular length from Condylion to Gnathion (mm).
- *Co*-*Go*: length of mandibular ramus from Condylion to Pogonion (mm).
- *Go*-*Pg*: length of mandibular body from Gonion to Pogonion (mm).
- *ArGoMe*: Gonial angle (Articulare-Gonion-Menton) (degrees).
- *IMPA*: lower incisor to mandibular plane angle (degrees).

We adopted the same method described in^16^ to follow the Sagittal Skeletal Imbalance (SSI) during the growth process, as the difference between *Co*–*Gn* and *Co*–*A*, calculated both in *T*_1_ and in *T*_2_ for all 91 longitudinal Class III subjects^20^. To evaluate the progression of imbalance, the individual SSI of each Class III subject was compared, with the SSI standard values in a normal population (matched for age and gender) derived from the cephalometric atlas by Bhatia and Leighton^21^. “Good growers” were defined those subjects who approached normal values during the growth process (*T*_2_–*T*_1_ change), while “bad growers” were defined as those subjects who showed an increase of the difference with respect to normal values during *T*_2_–*T*_1_ change. Out of the 91 subjects, 28 (30.8%) were found to be good growers, 63 (69.2%) bad growers. A rigorous statistical analysis of the distributions and correlations of the used variables is out of the scope of this paper, but it can be noted from the histograms in Fig. 4. that the ages are roughly distributed with a prevalence of younger subjects, while the rest of the variables are bell-shaped. For a more detailed analysis of the population the reader is referred to *Auconi et al*. previous work^16^.

### Statistical Analysis

The longitudinal data at *T*_1_ were analysed with the following steps: 1) Classification Trees; 2) Regularized Logistic Models (LASSO); 3) Transductive Learning approach (Semi-supervised Learning); 4) Boosting; 5) Feature Engineering. A logical scheme of this sequence of operation can be found in Fig. 5.

#### Classification Trees

A Classification Tree is a combination of mathematical and computational techniques that can aid in the description, categorisation, and generalization of a given set of data, to help identifying a strategy that most likely will reach a goal^3,6,11^. In the present study, Classification Trees were applied with the aim of identifying the most important predictors (among the 11 cephalometric variables at *T*_1_) for the good or bad craniofacial growth (categorical dependent variable). Trees were produced using the Python library *scikit*-*learn*^3,22^.

#### Regularized Logistic Models (LASSO)

Logistic regression can be used to fit a predictive model of the relationship to an observed data set of binary decisions Y (dependent) and numerical observed variable *X* (independent). Logistic regression can be applied to model the dependence between *X* and *Y* as a posterior probability function. The observed variables form a linear combination with suitable coefficients, and the weighted sum is combined with a nonlinear function (the logistic) to obtain a value bounded between 0 and 1. To improve the generalisation error and interpretability, especially when there are limited samples, the model’s coefficients can be penalised to keep them as small as possible (“regularisation”). This approach can be accomplished by imposing an upper bound on the norm of the coefficient’s vector: the most common choices are the *L*^2^ norm, which constraints the sum of their squares, and the *L*^1^ norm, which pose a limit on the sum of their absolute values. In this framework the LASSO (Least Absolute Shrinkage and Selection Operator) uses a *L*^1^ penalty assumption itself. It can be proven that while the *L*^2^ norm just shrinks the regression coefficents, the *L*^1^ norms forces some of them so be exactly zero. This can be seen easily with a geometrical intuition on cartesian plane: a sum of squares penalty costrains the coefficients to stay in a disk shaped area, while a sum of absolute values forces them to stay in a diamond shaped area. In a higher dimensional space, the diamond becomes a rhomboid. These kind of shapes have many corners, so when the coordinate descent algorithm that minimize the loss function hits one of them, the coefficents corresponding to that coordinate becomes zero^23^. Thus, the advantages of LASSO are both easier interpretation of the fitted model (sparsity) and computational convenience (convexity). The regularised logistic classification model was performed using the Python library *scikit*-*learn*.

#### Transductive Learning (TL) Approach (Semi-Supervised Learning)

In many real-world scenarios concerning predictions it often is easy to collect large amounts of unlabeled (cross-sectional) data, while it is much harder to assign labels that requires careful work by a domain expert. In orthodontics typically it is easy to collect cross-sectional data, whereas following a patient longitudinally and assigning a label at the end of the observation period is much more difficult. In the last twenty years, several useful algorithms have been developed to learn from both cross-sectional and longitudinal labelled data, such as Semi-Supervised Learning, or Transductive Learning^11,12,14,15^. To exploit the values from cross-sectional sample, an algorithm based on Graph theory^24–27^ was used with the support of the implementation offered by the Python library *scikit*-*learn*. Each cephalometric observation was considered a vertex on the graph where an appropriate similarity measure (expressed by a radial basis kernel function) was defined among observations; this measure then was used to determine the weights related to the edges connecting each observation with the others. An iterative process by which information about true labels is spread between neighbours then was carried out until convergence or some stopping criterion. In this study the stopping criterion was chosen in order to spread the label information until the class balance in the cross-sectional sample was the same as in the longitudinal-sample, as this was the only reasonable assumpion to work with. For further mathematical and algorithmic details, the reader is referred to Chapelle *et al*.^12^ and Zhu^15^. The parameters of the algorithm, such as the radial basis function bandwidth, have been chosen with the same rationale as the stopping criterion cited above. This approach allows exploiting the cross-sectional dataset by augmenting the sample size without making use of oversampling techniques or completely “synthetic patients”^28^. A comparison was carried out between simple linear classifiers, such as logistic regression (LASSO) and Decision Tree boosting algorithm^13,25–28^. While simple linear models or a single Decision Tree offer great interpretability, these statistical approaches may fail to learn complex nonlinear relationships between input and output data^28,29^.

#### Boosting

Every predictive model has its strength and weaknesses. If one creates lots of models in lots of different ways and then combine them together, the “ensemble” of models uses several learning algorithms to obtain better predictive performance. Ensembles tend to yield better results when there is a significant diversity among the models^22,25–27^. The key concept of Boosting is to train the predictive algorithms in sequential rather than in parallel way. Every datum is weighted, and each new classifier is trained by giving more weight to previously misclassified data. In this way, the combination results are more efficient and the errors tend to decrease. This procedure allows to use very weak classifiers that produce a very strong classifier through Boosting^22^). The reweighting procedure is guided by gradient descent on the logistic loss function. The number of trees in the model, which can be seen as the number of iterations of the procedure, is a parameter to be chosen with cross validation as all the other parameters of the trees construction. This has to be done as, in contrast with other ensembles of trees such as random forests^30^, adding too many trees can lead to overfitting. Boosting can be interpreted as an optimisation algorithm that belongs to a family of Machine Learning procedures that converts weak predictive analytics to strong ones. The most common implementation of Boosting is AdaBoost^13,22^. The Boosting method utilised in this study belongs to the family of Gradient Boosting, and a very efficient implementation is offered by the XGBoost library^26^.

#### Feature Engineering

In Data Mining applications a common way to improve accuracy is combining existing variables to create new ones. Features that look irrelevant in isolation may be relevant in combination. A simple powerful procedure, when using linear classifiers to introduce non-linearity terms, is to add polynomial features, such as squares or other given functions of the existing variables, or multiplications between feature pairs, often called interaction terms. Linear combinations also can be used, with careful attention when considering models that suffer from collinearity. This process often is called Feature Engineering (FE)^10,22^. For the Data Mining practitioner, the guidance in FE application is dictated by prior knowledge from a domain expert. FE process produces a new list of variables to be used. We added the age at *T*_1_ in the data set, and we aggregated two differences *GoGn*–*SN*, *SNB*–*SNA* as proxy of horizontal maxillomandibular imbalance, and a sum of *ArGoMe* + *PPMP* + *PPSN* angles as a proxy of vertical imbalance. To reduce the clutter in the ranking of variable importance, some highly correlated variables were removed, this operation was guided by prior domain knowledge about craniofacial growth.

## Results

**Figure 2:**
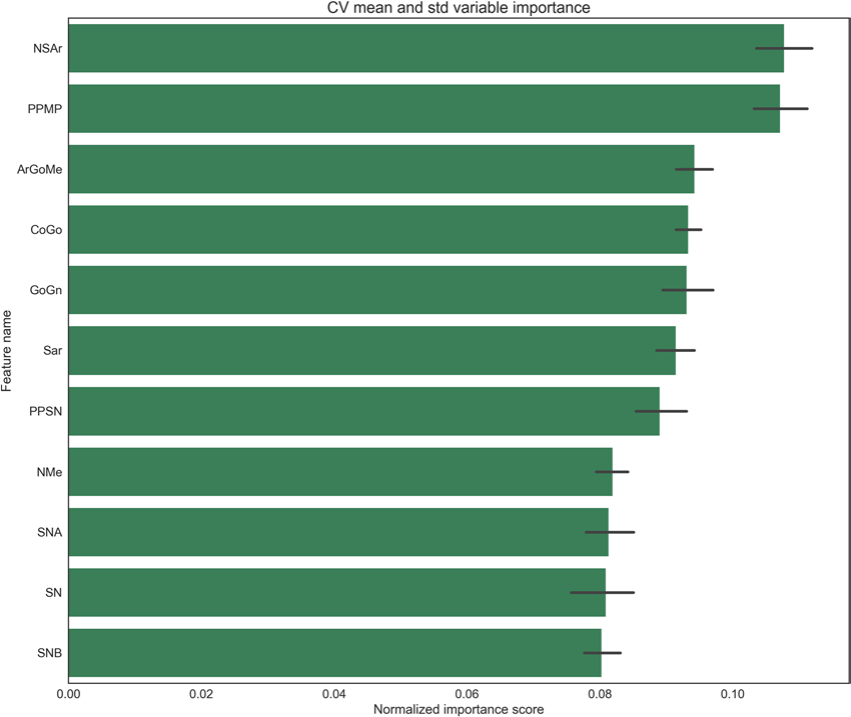
On the x-axis: Normalized importance ranking of cephalometric variables in predicting good/bad Class III growers. The ranking was obtained by averaging the information gain obtained through splitting on a specific variable across all trees, and then across all the folds of the K-fold cross-validation (K = 10). Error bars corresponding to the standard deviation across the folds is reported. It can be noted that, while significant, the error is not large enough to completely change the ranking.

**Figure 3:**
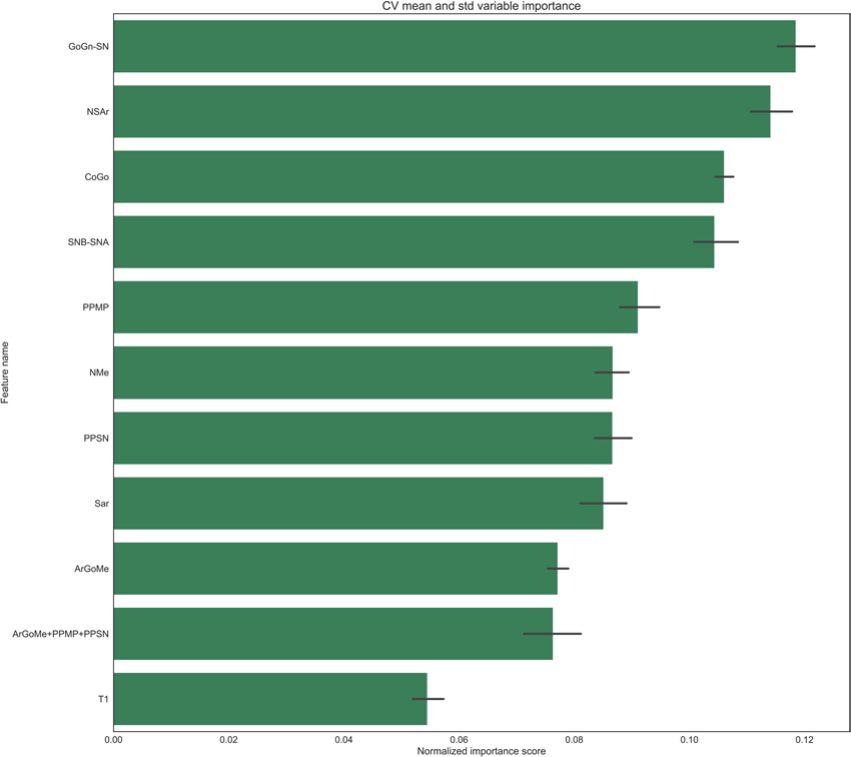
On the x-axis: Normalized importance ranking of cephalometric variables in predicting good/bad Class III growers. The ranking was obtained by averaging the information gain obtained through splitting on a specific variable across all trees, and then across all the folds of the K-fold cross-validation (K = 10). Error bars corresponding to the standard deviation across the folds is reported.

By using a single Classification Tree on cephalometric data of 91 longitudinal Class III subjects, we obtained an accuracy score of 63%, as occurred in the previous work of Auconi *et al.*^16^. Next, using the combination of TL preprocessing, a gradient Boosting classifier, and a logistic model LASSO, we observed improved accuracy scores from 63% to 78% (See Table 1). To ensure a fair comparison, the input data to the logistic model was standardized (zero mean, unit variance), while input data for boosting were the original ones. This is done because LASSO can suffer from numerical problems when the dynamic range of the variables is very different and in this case it spans two orders of magnitude. Moreover without standardization the resulting coefficents would depend on the unit of measure of the variables, making interpretation harder. On the other hand the tree-based boosting procedure is independent from the features’ scaling, and it does not suffer for this numerical problems. The *f*_1_ score is reported along with accuracy, precision, and recall in Table 1. The *f*_1_ score is the harmonic mean of precision (positive predictive value, the fraction of relevant instances among all the retrieved instances) and recall (the fraction of relevant instances that have been retrieved over the total amount of relevant instances). A mean ROC curve across the folds of the 10-fold cross validation is reported in Fig. 6 for the best found model (GB with TL and FE). This curve, built by varying the probability threshold used to decide for “Good” or “Bad” classes, allow the practioner to further tune the predictions to be more or less conservative with false negatives as needed. These results increased the ability to predict patients at risk of unfavorable growth reported in^16^, and offered as a side-product a classification by TL on the overall unlabelled cross-sectional dataset. Moreover, the procedures allowed the estimation of the generalization error using a 10-fold cross validation. In addition, the results confirmed that a simple linear model provided a modest accuracy score of 62%. To assess statistically the significance of the test, a Welch test has been performed on the *K* = 10 outcomes to check the mean equality hypothesis. The test provides a p-value < 0.001. The variable’s importance ranking (Fig. 2) obtained through the Boosting procedure can be used by the practicing orthodontist to explore the importance of the variables in affecting the model predictions. The variable’s importance ranking (Fig. 2) obtained through the Boosting procedure can be used by the practicing orthodontist to explore the importance of the variables in affecting how the model uses the variables to make its predictions. The practioner then can reason about these rankings and verify if they have a match with his/her experience and the literature. As far as we, as practioners, can say not only *PP*–*MP* and *SNA* angles, but also *NSAr* and *S-N* starting values were important features in differentiating the quality of craniofacial growth among Class III subjects. The Feature Engineering procedure obtained by the aggregation of some cephalometric measures to make them more expressive about the risk of maxillomandibular imbalance produced a further increase in accuracy of the model (up to 83%; Table 2). The same procedures as in the Table 2 was carried out for both the Gradient Boosting classifier and the Logistic *L*^1^ (LASSO). After FE, a new ranking of variables was obtained (Fig. 3). The horizontal skeletal imbalance *GoGn*–*SN* (Go-Gn minus S-N) was another important finding in characterising growth. Class III subjects showing early maxillomandibular horizontal imbalance tend not to recover the gap during the growth process. The little predictive information importance of the aggregation of three angles of vertical imbalance *ArGoMe*, *PPSN*, and *PPMP* was noteworthy, while the relevance of the single *PP*–*MP* angle was confirmed. Likely, among the skeletal angles of vertical imbalance, the signal of unfavourable growth is nested in the latter angle. The orthodontic practitioner can find the predictive algorithm derived from this study and free to use for their patients at the following webpage http://malocclusion3.pythonanywhere.com/.

**Table 1:** Comparison of various configurations of experiments on the K-fold cross-validation (K = 10), mean and standard deviation (SD) across the folds are reported.

	Mean cv accuracy	SD cv accuracy	Mean cv f1 score	SD cv f1 score	Mean cv precision	SD cv precision	Mean cv recall	SD cv recall
LASSO	0.626	0.123	0.536	0.165	0.433	0.142	0.710	0.210
LASSO (TL)	0.620	0.186	0.506	0.244	0.450	0.253	0.666	0.365
LASSO (TL + FE)	0.684	0.128	0.553	0.159	0.505	0.117	0.666	0.258
GB	0.648	0.187	0.445	0.297	0.444	0.307	0.495	0.336
GB (TL)	0.782	0.147	0.628	0.287	0.627	0.305	0.650	0.300
GB (FE + TL)	0.834	0.088	0.710	0.164	0.768	0.201	0.700	0.221

Gradient Boosting is more accurate than Logistic L1. The higher f1 score shows that it also balances errors in a better way. While through direct inspection of the logistic model a clear interpretation of how the prediction can be made, the accuracy and balance of the model is much poorer. The addition of TL reduces the standard deviation of the results of the GB algorithm.

**Table 2:** Mean and standard deviation (Std) of the sample grouped by age of the patient.

Age	ArGoMe	CoGo	NMe	NSAr	PPMP	PPSN	Sar	SN	SNA	SNB	GoGn
7	130.54 (5.50)	46.64 (3.68)	105.19 (5.87)	120.91 (5.47)	26.92 (4.47)	7.95 (3.58)	29.17 (2.65)	68.04 (3.47)	80.30 (3.48)	79.45 (3.35)	72.43 (4.81)
8	129.78 (6.04)	48.64 (3.97)	108.86 (6.28)	122.09 (5.01)	26.46 (4.95)	8.36 (4.95)	30.30 (2.88)	69.50 (3.62)	79.72 (3.57)	79.05 (3.46)	74.69 (4.63)
9	129.24 (6.36)	49.56 (4.12)	110.26 (6.73)	121.42 (4.90)	26.38 (4.96)	8.34 (3.11)	30.66 (3.22)	69.56 (3.81)	80.06 (3.39)	79.46 (3.32)	75.43 (4.69)
10	130.52 (6.33)	50.41 (4.59)	112.52 (5.95)	121.45 (5.04)	26.10 (5.00)	7.90 (2.86)	31.96 (2.76)	70.52 (3.73)	81.14 (3.94)	80.61 (3.61)	77.21 (4.92)
11	130.57 (5.17)	52.89 (4.74)	117.85 (6.66)	122.66 (4.68)	27.91 (5.40)	8.65 (2.86)	32.22 (2.75)	71.01 (3.82)	80.00 (3.85)	79.63 (3.25)	80.20 (4.85)
12	130.56 (6.73)	54.66 (6.42)	120.30 (7.51)	122.47 (4.75)	27.16 (6.18)	9.05 (3.75)	32.62 (3.70)	72.42 (3.75)	80.29 (4.16)	79.99 (3.73)	82.05 (6.21)
13	129.45 (6.09)	55.69 (5.75)	122.32 (8.18)	122.36 (5.49)	26.66 (4.88)	8.79 (2.92)	33.91 (3.60)	72.10 (3.62)	80.65 (3.56)	80.36 (3.08)	82.77 (5.88)

**Figure 4:**
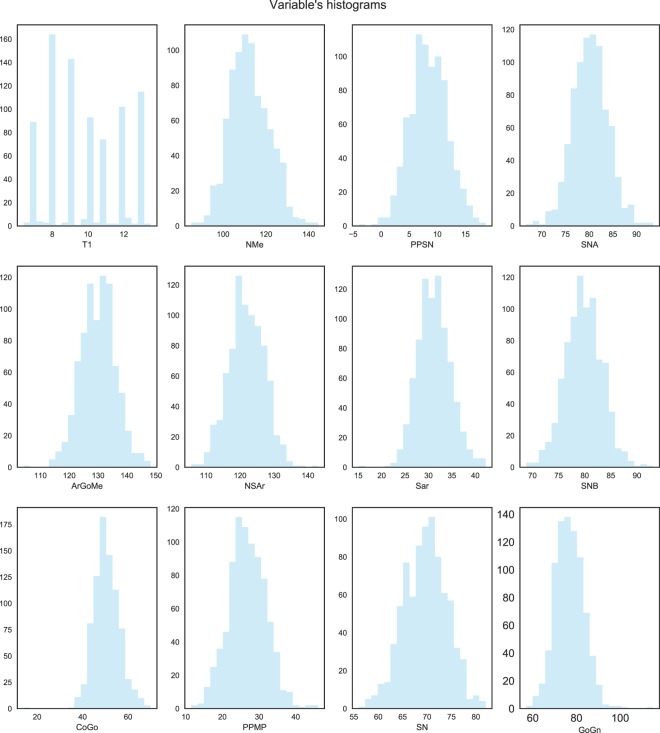
Histograms of the used variables.

**Figure 5:**
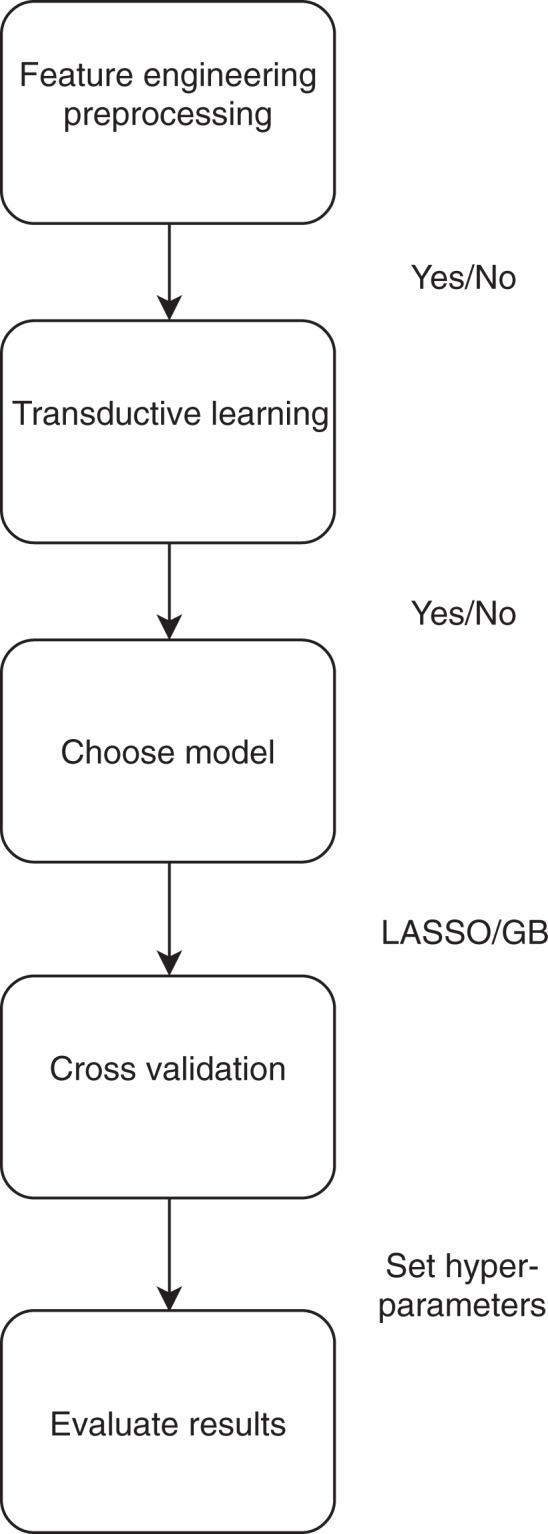
Logical scheme of the analysis sequence.

**Figure 6:**
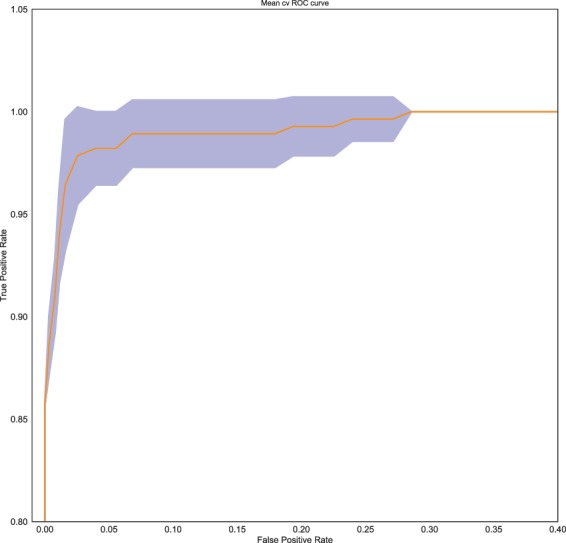
GB (with TL and FE) Mean ROC curve (with the standard deviation represented by the blue shadow) across the k-fold cross-validation (K = 10).

## Discussion

**Figure 7:**
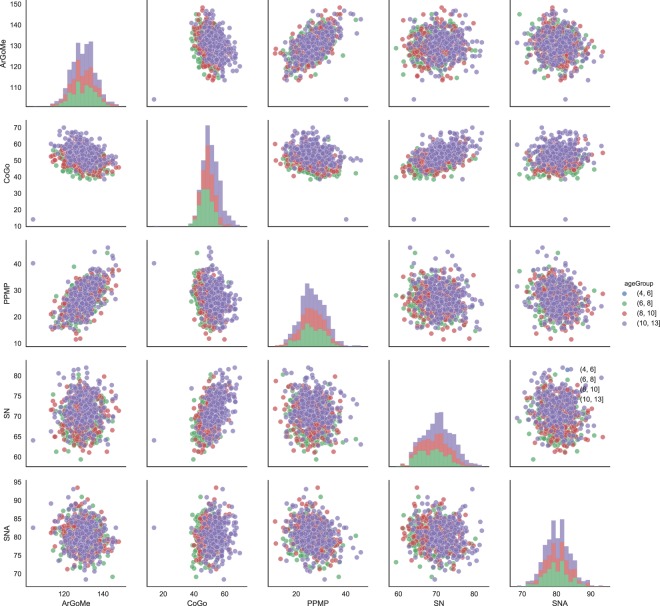
Histograms and scatterplots of some of the variables by age group.

Biomedical researchers and practitioners always have dreamed of prognostic predictive models that bind the observable reality with its mechanism of origin, especially if made up of a small number of easily identifiable parameters. In order to progress, a medical discipline must have data and reasoning commensurate with the complexity of the phenomena of interest. Data Mining and Machine Learning are disciplines of data science that aim to uncover unknown relationships and patterns from datasets whenever this is possible^4,5,28,29,31^. From the beginning, these approaches to medicine have been sources of controversies: the main doubts concerned to what extent medical knowledge could be truly compressed and automatically coded into rules, and whether these aggregates could lead to an unavoidable amount of over-simplification^4,5^. Actually, early techniques seemed to have modest practical value in handling the complexity of biomedical features appropriately, and in recognizing clinical variation of a disease, such as change in severity over time. It was difficult to capture relevant diagnostic information in strict rule-based systems with an acceptable degree of reliability, or to cope with rules interacting with many other rules in a complex and unpredictable way^32^. These concerns have been overcome due the construction of reliable innovative models such as Decision Trees, Bayesian networks, and Boosting procedures. While it must be remembered that inference is always done in a probabilistic setting, even more and more realiable algorithms and sophisticated validation procedures can still be failing^6,9,10^. When considering scenarios in clinical orthodontics, it is easy to claim that hypotheses about causal laws that affect the onset and the progression of disharmonies are probabilistic, and hence fallible^33,34^. In current clinical practice, the orthodontist’s judgments are constrained by limited and subjective experience, incomplete knowledge, and oversimplified taxonomy of malocclusions that underestimate relevant individual qualitative aspects of disharmonies. Causes can be reversed, cause and effects are interchangeable, treatment effects can be the result of multiple causes, and the learned cause does not necessarily imply to be the only cause^18^. Consequently, orthodontic researchers and practitioners had to develop a kind of “inexact reasoning” based on a set of hypotheses concerning the probabilistic relationships between combinations of early orthodontic signs, malocclusion progression, treatment decisions, and outcomes^29,31,34^. Practicing orthodontists must consider questions like: “Can I find correlations between specific craniofacial characteristics and patterns of future growth?”, “What are the most influential skeletal sites useful for my treatment purposes?”, and “What co-occurrence of dentoskeletal characteristics make my treatment difficult?”. With the progression of treatment and better understanding of the characteristics of the patient, paradoxically these questions become more complex and difficult to answer. It is within this context that the cognitive tasks of ML could play a role in orthodontic diagnosis and prognosis. At the beginning, various algorithms are applied to generate a prediction model to find relationships that correlate with events and non-events^3^. The success of the predictive model derives from the accuracy of this process; there often are several parameters that need to be set. The first stage of the prognostic process involves making an initial judgment about whether a Class III patient is likely to have a bad/good growth progression, based on previous experience and knowledge of literature. Unlike to the medical practitioner, the orthodontist does not have the option of exploiting a post-test probability (such as, for example, an exercise stress-test for the coronary disease). Predictive models, by their very nature, are based on the past and assume that the patterns of the past are repeated. In orthodontic discipline this assumption is obviously problematic. In the present work we re-analysed the same longitudinal data set of Auconi *et al.*^16^, exploiting the combination with a cross-sectional data of 728 untreated Class III subjects (see Table 3 for the complete correlation matrix of the variables considered) through a TL learning procedure. Subsequently, we submitted the data to a B and FE procedures. This sequence increased the predictive capability of adverse growth from 62% to 83%, through a further deepening of the data structure and a careful matching between longitudinal and cross-sectional cephalometric characteristics (see in Fig. 7 the histograms and scatter plots by age group). Despite the increasing popularity of several predictive models from Machine Learning applied to medicine, some statisticians have argued against the superiority of these sophisticated methods in comparison to early easier methods such as Discriminant analysis (the “illusion of progress”)^35^. Moreover, as already has happened in other biomedical fields, also in orthodontic discipline there is the concern that the allure of novel computational investigative methods could lead to misuse of information and inconsistent clinical analyses, further distracting the clinician from properly looking at the morphological and functional individuality of the patient^35–39^. This work shows that constructing an appropriate predictive model of Class III progression, and providing a more in-depth understanding of critical issues related to dentoskeletal disharmonies during the growth process can support the daily work of clinical orthodontist, reducing the bias of subjective judgment.

**Table 3:** Correlation matrix between the variables.

	ArGoMe	CoGo	NMe	NSAr	PPMP	PPSN	Sar	SN	SNA	SNB	GoGn
ArGoMe	1	−0.22	0.16	0.07	0.56	0.10	−0.09	0.04	−0.11	−0.22	−0.28
CoGo	−0.22	1	0.62	0.04	−0.25	−0.01	0.50	0.50	0.11	0.23	0.54
NMe	0.16	0.62	1	0.09	0.32	0.21	0.56	0.52	−0.14	−0.21	0.62
NSAr	0.07	0.04	0.09	1	−0.05	0.38	−0.05	−0.08	−0.41	−0.43	0.02
PPMP	0.56	−0.25	0.32	−0.05	1	−0.21	−0.09	−0.11	−0.22	−0.42	−0.05
PPSN	0.10	−0.01	0.21	0.38	−0.21	1	−0.16	0.06	−0.35	−0.47	0.01
Sar	−0.09	0.50	0.56	−0.05	−0.09	−0.16	1	0.31	0.11	0.16	0.45
SN	0.04	0.50	0.52	−0.08	−0.11	0.06	0.31	1	−0.13	−0.03	0.52
SNA	−0.11	0.11	−0.14	−0.41	−0.22	−0.35	0.11	−0.13	1	0.79	0.17
SNB	−0.22	0.23	−0.21	−0.43	−0.42	−0.47	0.16	−0.03	0.79	1	0.28
GoGn	−0.28	0.54	0.62	0.02	−0.05	0.01	0.45	0.52	0.17	0.28	1

## Data Availability

The orthodontic practitioners can find the predictive algorithm derived from this study and free to use for their patients at the following webpage http://malocclusion3.pythonanywhere.com/.
